# Neuroinflammatory Responses and Blood–Brain Barrier Injury in Chronic Alcohol Exposure: Role of Purinergic P2X7 Receptor Signaling

**DOI:** 10.21203/rs.3.rs-4350949/v1

**Published:** 2024-05-08

**Authors:** Namdev S. Togre, Naveen Melaka, Priyanka S. Bhoj, Nikhita Mogadala, Malika Winfield, Jayshil Trivedi, Deborah Grove, Sudhir Kotnala, Slava S Rom, Uma Sriram, Yuri Persidsky

**Affiliations:** Temple University; Temple University; Temple University; Temple University; Temple University; Temple University; Temple University; Temple University; Temple University; Temple University; Temple University

**Keywords:** CIE, Blood-brain barrier, ATP, P2X7R, Extracellular vesicles

## Abstract

Alcohol consumption leads to neuroinflammation and blood–brain barrier (BBB) damage, resulting in neurological impairment. We previously demonstrated that ethanol-induced disruption of barrier function in human brain endothelial cells was associated with mitochondrial injury, increased ATP and extracellular vesicle (EV) release, and purinergic receptor P2X7R activation. Therefore, we aimed to evaluate the effect of P2X7r blockade on peripheral and neuro-inflammation in EtOH-exposed mice.

In a chronic intermittent ethanol (CIE)-exposed mouse model, P2X7R was inhibited by two different methods: Brilliant Blue G (BBG) or gene knockout. We assessed blood ethanol concentration (BEC), plasma P2X7R and P-gp, number of extra-cellular vesicles (EV), serum ATP and EV-ATP levels. Brain microvessel gene expression and EV mtDNA copy numbers were measured by RT2 PCR array and digital PCR, respectively.

A RT2 PCR array of brain microvessels revealed significant upregulation of proinflammatory genes involved in apoptosis, vasodilation, and platelet activation in CIE-exposed animals, which were decreased 15–50-fold in BBG-treated CIE-exposed animals. Plasma P-gp levels and serum P2X7R shedding were significantly increased in CIE-exposed animals. Pharmacological or genetic suppression of P2X7R decreased P2X7R shedding to levels equivalent to those in control group. The increase in EV number and EV-ATP content in the CIE-exposed mice was significantly reduced by P2X7R inhibition. CIE mice showed augmented EV-mtDNA copy numbers which were reduced in EVs after P2X7R inhibition or receptor knockout.

These observations suggested that P2X7R signaling plays a critical role in ethanol-induced brain injury. Increased eATP, EV-ATP, EV numbers, and EV-mtDNA copy numbers highlight a new mechanism of brain injury during alcohol exposure via P2X7R and biomarkers of such damage. In this study, for the first time, we report the *in vivo* involvement of P2X7R signaling in CIE-induced brain injury.

## Introduction

Alcohol abuse and its detrimental effects on the central nervous system (CNS) have long been recognized as significant public health concerns. Excessive alcohol consumption is listed as one of the leading risk factors for population health, disease, disability, and death worldwide. A recent report, “World Alcohol and Health Situation,” from the World Health Organization indicated that more than 3 million deaths attributed to alcohol consumption correspond to one death every 10 seconds [1].

Several studies have reported that excessive alcohol consumption causes damage to various organs [2, 3, 4]. The key mechanisms underlying alcohol-induced neurotoxicity involve neuroinflammation and blood-brain barrier (BBB) disruption, which contribute to neuronal damage and dysfunction [3, 5]. Loss of BBB function is associated with increased permeability and downregulated expression of key proteins in the BBB [6, 7]. Disruption of BBB integrity leads to the infiltration of peripheral immune cells, cytokine release, and subsequent neuroinflammatory responses, exacerbating neuronal injury [8, 9].

Purinergic receptor-mediated signaling is essential in the CNS for maintaining physiological neural cell function and has emerged as a crucial modulator of neuroinflammation and BBB function [10, 11]. Among purinergic receptors, the purinergic receptor P2X7 (P2X7R) is involved in inflammatory processes and cell death cascades [12]. Additionally, its association with BBB disruption is of interest [13]. Adverse cellular conditions, such as stress and cellular damage, lead to an increase in extracellular ATP (eATP) concentrations, which act as damage-associated molecular patterns (DAMPs); supraphysiologic ATP concentrations are responsible for P2X7R activation. *In vitro* chronic alcohol exposure of human macrophages results in the activation of the P2X7R-mediated Nod-like receptor pyrin domain containing 3 (NLRP3) inflammasome pathway, which causes the secretion of interleukin 1 beta (IL-1β) [14]. Moreover, ethanol (EtOH)-dependent P2X7R overactivation causes alcohol-induced BBB damage with increased levels of the proinflammatory cytokines IL-1β, tumor necrosis factor alpha (TNF-α), and interleukin-6 (IL-6) in mice [15, 16]. In a series of investigations, our laboratory revealed the effects of EtOH exposure on brain microvascular endothelial cells (BMVECs) and revealed a compelling link between substance exposure and dysregulation of purinergic signaling pathways. EtOH-exposed BMVECs showed the mito-stress and enhanced eATP release, which were prevented by P2X7R antagonist [17–19].

Extracellular ATP stimulates P2X7R, which triggers extracellular vesicle (EV) shedding [20, 21]. EVs are cargo-carrying cell-derived vesicles which can communicate between originating and recipient cells. Several reports have stated the changes in cargo composition based on host cell health status [22–24]. P2X7R activation can change EV proteome and may be involved in the propagation of inflammation. EVs carry cytokines, various mRNAs, lipids, and ATP molecules [22, 23, 25]. Chronic EtOH exposure increases levels of proinflammatory molecules in EVs [25–27]. Studies have also detected the presence of mitochondrial DNA (mtDNA) fragments with DAMP-like properties in EVs isolated after chronic EtOH exposure [27, 28].

Several studies have reported the undeniable role of P2X7R signaling in BBB injury *in vitro* [18, 29–33]. However, the precise mechanisms underlying P2X7R-mediated effects in alcohol-induced neuroinflammation *in vivo* remain incompletely understood. In this study, we hypothesize that blocking of P2X7r signaling either by administration of BBG (P2X7r blocker) or P2X7r genetic deletion (P2X7r^−/−^) will reduce neuroinflammation and BBB injury in chronic EtOH-exposed mice.

In this study, we found that pharmacologic or genetic inhibition of P2X7R significantly decreased the levels of upregulated brain proinflammatory cytokines, circulating P2X7R, serum ATP levels, EVs, EV-ATP, and EV-mtDNA fragments in a mouse model of chronic intermittent exposure (CIE) to EtOH. Furthermore, the genes involved in apoptosis, vasodilation, and platelet activation, which were significantly upregulated in the brain microvessels of alcohol-exposed mice, were downregulated in CIE-exposed mice treated with the P2X7R inhibitor.

## Materials and Methods

### Animals

Wild-type and P2X7r^−/−^ C57BL/6 (B6.129P2-*P2rx7*^*tm1Gab*^/J, stock no. 005576) mice (male, 16–17 weeks old) were obtained from Jackson Laboratories, Bar Harbor, ME. To achieve statistical significance, 5–15 mice in each experimental group were used. In the pharmacologically P2X7R-inhibited cohort, 7 mice were used in the air control group, 5 in the BBG-treated CIE-unexposed, 7 in the CIE-exposed, and 8 in the BBG-treated-CIE-exposed group. For P2X7R knockout cohort, 8 mice were used in the wild-type air control group, 6 in the P2X7r^−/−^ CIE-unexposed, 15 in the wild-type CIE-exposed, and 15 in the P2X7r^−/−^ CIE-exposed group. The mice were housed five per cage with food and water available *ad libitum* (12-h light-dark cycle). All *in vivo* experiments were approved by the Temple University Institutional Animal Care and Use Committee in accordance with guidelines based on the National Institutes of Health (NIH) Guide for Care and Use of Laboratory Animals and the Animal Research: Reporting *in Vivo* Experiments (ARRIVE) guidelines (www.nc3rs.org.uk/arrive-guidelines; accessed on March 19, 2022).

### CIE and BBG injections

**Figure 1:**
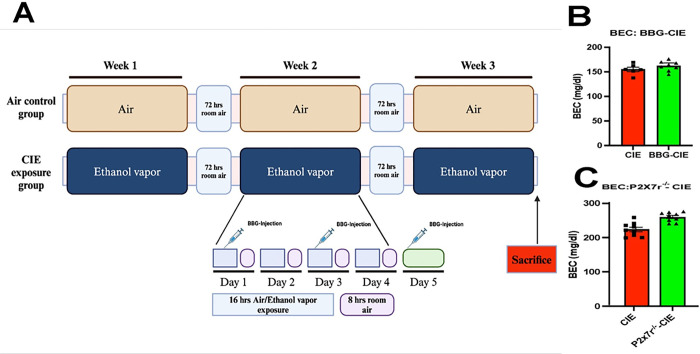
Schematic of the experiment and blood ethanol concentrations (BECs). **(A)** CIE exposure paradigm. Created with BioRender.com **(B and C)** BECs were assessed to ensure that pathophysiologically relevant ethanol levels were obtained at the end of the experiment. The mean BECs were 154.98 ± 10.70 mg/dl, 161 ± 10.19 mg/dL, 223.59 ± 20.15 mg/dL, and 259.73 ± 13.73 mg/dL in the CIE, BBG-CIE, CIE and CIE-P2X7r^−/−^ groups, respectively.

A mouse model of CIE exposure was developed as described previously [34–36] with the following modifications. All the mice in the CIE-exposed groups were exposed to continuous ethanol vapor for 16 h, followed by 8 h in room air each day for four days a week (1 cycle; Fig. 1A). The exposure cycle was repeated three times. Before placing the mice in ethanol vapor, an intraperitoneal (i.p.) injection of an alcohol dehydrogenase inhibitor, pyrazole (P56607–5G; Merck, USA, 85 mg/kg), and a loading dose of 1.0 g/kg ethanol (32801; Decon labs Inc.) (20% w/v) in 0.9% saline were given to initiate and maintain stable ethanol intoxication in the CIE-exposed mice [37]. The mice in the air control group were injected with 85 mg/kg pyrazole in saline. To deliver ethanol vapor, 190-proof ethanol was volatilized, mixed with fresh air at a rate of 10 L/min, and then pumped into the ethanol inhalation chamber. At the end of every cycle, a 2 mL air sample was drawn through a port in the chamber door to measure the amount of ethanol present in the chamber. Mice from the BBG- and BBG-CIE-exposed groups were injected i.p. with 45 mg/kg mouse body weight BBG ([ab120389; Abcam] in 100 μL of 0.9% saline) to inhibit P2X7R *in vivo*.

### Blood ethanol concentrations

At the end of the experiment, blood ethanol concentrations (BECs) were measured [38]. Blood samples were collected in 0.5 M EDTA (pH 8) through the submandibular vein punch immediately after removal of the mice from the ethanol vapor chamber. The isolated serum samples were subjected to a spectrophotometric enzymatic assay (ECET-100TM Ethanol Assay Kit; BioAssay Systems, San Francisco, USA).

### Brain Microvessel Isolation

Mouse brain microvessels were isolated using an earlier published protocol with some modifications. [19, 39, 40]. In brief, the mice were perfused with saline, and the brains were harvested. All the following steps were carried out on ice. Following a wash in PBS and removal of the cerebellum, meninges, and large superficial blood vessels, the right hemisphere of the brain was homogenized in 1 mL of ice-cold Hank’s balanced salt solution (HBSS) using a Dounce homogenizer (357538; Grienger, Philadelphia, USA) (0.25 mm clearance). Overall, the resulting homogenate was centrifuged at 1000 × *g* for 10 minutes, and the pellet was resuspended in ~ 5 mL of cold 17.5% dextran and centrifuged for 15 minutes at 4400 × *g* at 4°C. Using a cut tip, the supernatant containing the myelin layer was removed, and the remaining pellet was resuspended in ~ 5 mL of HBSS containing 1% BSA. After the suspension was broken up using a 10 mL pipet in a Petri dish, it was passed through a 100 μm mesh nylon filter. The collected filtrate was passed through a 40 μm mesh nylon filter. The microvessels retained in the filter were collected by inverting the filter and rinsing it with 3 mL of HBSS containing 1% BSA. Finally, after centrifugation, the microvessels were collected and stored at −80°C for further processing.

### Gene expression profiling (qRT–PCR)

cDNA was synthesized from 300 ng of total RNA from microvessels using a High-Capacity cDNA Reverse Transcription Kit according to the manufacturer’s instructions. The synthesized cDNA samples were stored at −20°C for later use. Real-time PCR was carried out by using a QuantStudio^™^ 3 Real-Time PCR System (Thermo Fisher Scientific; Waltham, USA).

qRT–PCR was performed by using the Qiagen Mouse Endothelial Cell Biology RT2 Profiler PCR Array (PAMM-015Z) in combination with RT2 SYBR^®^ Green qPCR Mastermix (Qiagen, USA) according to the manufacturer’s recommendations [41, 42].

### Serum proinflammatory markers

Multiplex detection of serum proinflammatory markers was performed using the V-PLEX Proinflammatory Panel 1 Mouse Kit (MSD) (Cat No: K15048D-1; Meso Scale Discovery, Rockville, USA) according to the manufacturer’s instructions [43]. The assay allowed for the measurement of keratinocyte chemoattractant (KC)/human growth-regulated oncogene (GRO) (KC/GRO), TNF-α, interferon gamma (IFN-γ), IL-1β, interleukin-2 (IL-2), interleukin-4 (IL-4), interleukin-5 (IL-5), IL-6, interleukin-10 (IL-10), and interleukin-12 p70 (IL12p70). Data from V-PLEX Meso Scale experiments were analyzed based on standard curves included in the respective assays using MSD Discovery Workbench software (DISCOVERY WORKBENCH version 4.0.13).

### Mouse glycoprotein (P-gp) and P2X7R levels

Plasma P-glycoprotein (P-gp) levels were determined using a kit-based protocol according to the manufacturer’s instructions (MBS450526, MyBioSource, San Diego, USA). P2X7R ELISA was performed using a mouse purinergic P2X7r ELISA kit (Cat. No. E12339m-American Research Products, Waltham, MA, USA) with some modifications [17]. Circulating P2X7R levels were detected in serum samples collected at the end of harvest. The absorbance was measured at 450 nm using a microplate reader (SpectraMax^®^ M5).

### Plasma EV isolation and nanoparticle tracking analysis

EVs from plasma samples were isolated according to a kit-based protocol (cat. no. 4484450; Invitrogen, USA) [44]. Nanoparticle tracking analysis (NTA) of isolated EVs was performed using the NanoSight NS300 system fixed with a 488 nm laser (Malvern Technologies, Malvern, UK). Briefly, EV samples were diluted (1:500) in 1 mL of particle-free Milli-Q water (Milliporesigma, Burlington, USA) and injected into the NanoSight chamber using a 1 mL BD slip-tip syringe (Cat. No. 309659, Franklin Lakes, USA). Prior to running the samples, the machine was calibrated using 100 nm latex beads from Malvern (Cat. No. NTA4088). The data were analyzed by NTA 3.3.104 software [17, 45].

### ATP detection in serum and EVs

Extracellular ATP levels in serum samples and EV suspensions were measured using the Luminescent ATP Detection Assay Kit from Abcam (Cat. No. ab113849, Cambridge, UK) in accordance with the manufacturer’s instructions with a few modifications. The EV suspension was subjected to sonication to lyse and then centrifuged at 10,000 rpm for 5 min [17]. Serum samples (35 μL) or EV supernatants (50 μL) were added to a Corning^®^ black clear bottom 96-well plate (Cat. No. 3603, Corning, USA) along with the standards. A total of 50 μL of detergent was added to each well and incubated for 5 min at 600 rpm on an orbital shaker. Then, 50 μL of substrate was added to all the wells, followed by shaking at 600 rpm. The plates were covered and incubated in the dark for 10 min. Finally, luminescence was measured on an Infinite^®^ 200 M PRO (Tecan Austria GmbH).

### EV DNA isolation

To remove any DNA affixed to the EV surface, 100 μL of the EV suspension was treated with 10 U of DNase (LGC Biosearch Technologies, Cat. No. DB0715K, Hoddesdon, UK) for 20 min at 37°C. DNase activity was stopped by the addition of 10 μL of 10X DNase stop solution. Following further dilution with 100 μL of nuclease-free water (NFW), the resultant EV suspension was lysed at room temperature using 20 μL of proteinase K (Cat. no. 4485229, Thermo Fisher Scientific; Waltham, USA). The DNeasy^®^ Blood & Tissue Kit from Qiagen (Cat. no. 69506, Hilden, DE) was used to isolate DNA from this EV suspension [17, 46, 47].

### EV mtDNA Quantification by Digital PCR

The isolated EV-DNA was diluted to a working concentration of 1 ng/μL with NFW. Mitochondrial gene-specific Taqman^™^ probes for ATP8 [mt-ATP8] (Cat. no. 4331182 Mm04225236_g1), NADH dehydrogenase 2 [mt-ND2] (Cat. no. 4331182 Mm04225288_s1), cytochrome c oxidase subunit II [mt-COX2] (Cat. no. 4331182 Mm03294838_g1), and 16S ribosomal RNA [mt-RNR2] (Cat. no. 4331182 Mm04260181_s1) were used in this experiment [47] (Thermo Fisher Scientific; Waltham, USA). PCRs were performed using 2 μL of 5X Absolute Q^™^ DNA Digital PCR Master Mix (Cat. no. A52490), 2 μL of EV-DNA template (2 ng), 0.5 μL of FAM-Taqman^™^ probe, and 5.5 μL of NFW. A total of 9 μL of the above reaction mixture was loaded onto a QantStudioTMMAP16 Digital PCR plate (Cat. no. 10246917). Following the addition of 15 μL of QuantStudioTM Absolute QTM Isolation Buffer (Cat. no. A52730) to each sample, the wells were sealed using gaskets that were provided with the dPCR plates. The PCR for mtDNA dPCR was as follows: 10 min at 96°C, followed by 40 cycles of 5 s at 96°C and 15 s at 60°C. The QuantStudio^™^ Absolute Q Digital PCR System and QuantStudio dPCR software were used for DNA amplification, and the number of microchambers with successful mtDNA amplification was counted.

### Statistical analysis

The results are expressed as the mean ± SEM. The significance between the groups was assessed using Student’s t test. Multiple group comparisons were performed by one-way analysis of variance (ANOVA) with Tukey’s post hoc test [19]. Statistical analyses were performed utilizing Prism v10.2.1 (339) software (GraphPad Software Inc., La Jolla, CA). p ≤ 0.05 was considered to indicate statistical significance.

## Results

### BECs

Mice were exposed to ethanol vapors 4 days per week (16 h/day) to ensure that pathophysiologically relevant BECs were generated and maintained throughout the experiment. The observed BECs were 154.98 ± 10.70 mg/dL, 161 ± 10.19 mg/dL, 223.59 ± 20.15 mg/dL, and 259.73 ± 13.73 mg/dL in the CIE, BBG-CIE, CIE, and CIE-P2X7r^−/−^ groups, respectively (Fig. 1B and C).

### Effect of alcohol and P2X7R inhibition on gene expression in brain microvessels

**Figure 2:**
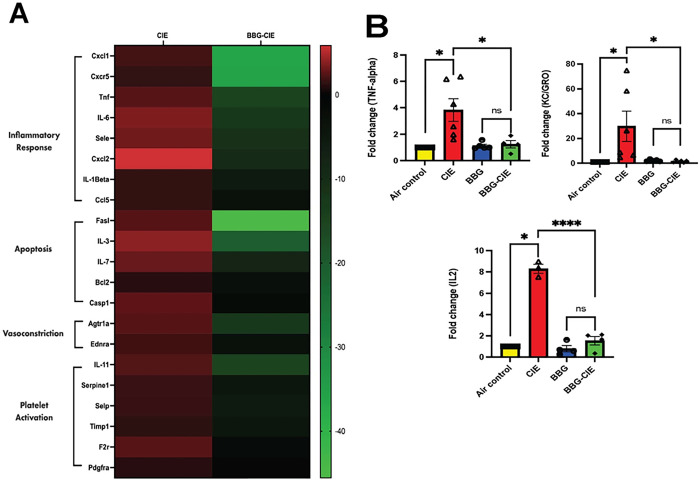
P2X7R inhibition downregulated the increase in the expression of genes involved in inflammation, apoptosis, vasodilation, and platelet activation in brain microvessels and serum cytokine levels in BBG-treated CIE-exposed animals. **(A)** Heatmap shows the upregulation of genes involved in inflammation, apoptosis, vasodilation, and platelet activation in the brain microvessels of CIE animals. BBG treatment led to significant downregulation of these genes. **(B)** Cytokine levels after CIE exposure were analyzed by MSD ELISA. The levels of the proinflammatory cytokines TNF-a, KC/GRO, and IL-2 were significantly lower in BBG-treated CIE-exposed animals than in CIE-exposed animals only. A two-tailed t test was used for the statistical analyses. The values are presented as the mean ± SEM;n = 3–7; *p <0.05, and **** p < 0.0001 compared with CIE-exposed mice.

Several studies have used brain microvessels to study the BBB and inflammation *in vitro* [48, 49]. CIE exposure significantly upregulated the expression profile of genes associated with inflammation (*cxcl1, il1b, cxcr5, tnf, il6, sele, cxcl2, and ccl2*), apoptosis (*fasl, il3, bcl2, casp1, and il7*), vasodilation (*ednra* and *agtr1a*), and platelet activation (*serpine1, selp, timp1, il11, f2r, and pdgfra*) in the brain microvessels of CIE-exposed animals. P2X7R inhibition significantly downregulated the CIE-induced neuroinflammatory response by 12–50-fold in the BBG-treated CIE-exposed group (Fig. 2A and Table 1).

### Modulation of serum cytokine levels by P2X7R inhibition

Analysis of serum cytokine levels using MSD ELISA revealed a significant increase (2–30-fold) in proinflammatory cytokine levels in CIE-exposed animals. A notable reduction in the serum levels of proinflammatory cytokines was observed after P2X7R suppression by BBG in CIE-exposed animals. Significant decreases in TNF-α, KC/GRO, and IL-2 levels were detected in BBG-treated CIE-exposed animals compared with CIE-exposed controls (Fig. 2B). Although not reaching statistical significance, the IL-1β, IFN-γ, and IL-5 levels also exhibited a decreasing trend in BBG-treated animals. A greater level of IL-10 was detected in BBG-treated CIE-exposed animals than in CIE-exposed animals (data not shown).

### P2X7R levels are increased in CIE mice

**Figure 3:**
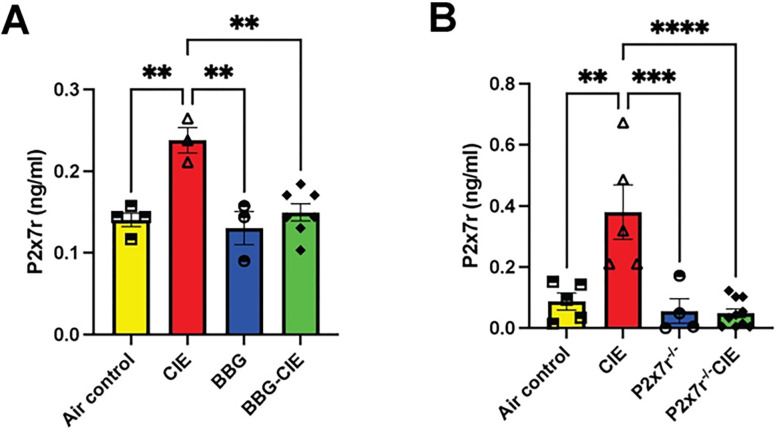
Serum levels of P2X7R upregulated by CIE were suppressed by receptor inhibition. **A)** BBG treatment and **B)** P2X7R^−/−^ resulted in significantly less P2X7R shedding in the serum than that in the serum of the CIE-exposed mice. One-way ANOVA followed by Tukey’s post hoc test was used for the statistical analyses; ** p ≤ 0.01, ***p ≤ 0.001, **** p < 0.0001 compared with CIE-exposed mice as controls. (n = 3–7, mean ± SEM).

P2X7R shedding has been implicated in chronic inflammation and neurodegenerative diseases (Jiang et al., 2022; Savio et al., 2018; Wang et al., 2017). Earlier in the *in vitro* study, we found enhanced P2X7R shedding after EtOH exposure [18]. *In vivo* CIE exposure increased serum P2X7R levels by 2–4-fold compared with the air control group. BBG treatment or P2X7R knockout significantly reduced P2X7R shedding in CIE-exposed mice (Fig. 3A and B).

### CIE-upregulated P-glycoprotein (P-gp) was not reduced by P2X7R inhibition or genetic knockout

**Figure 4:**
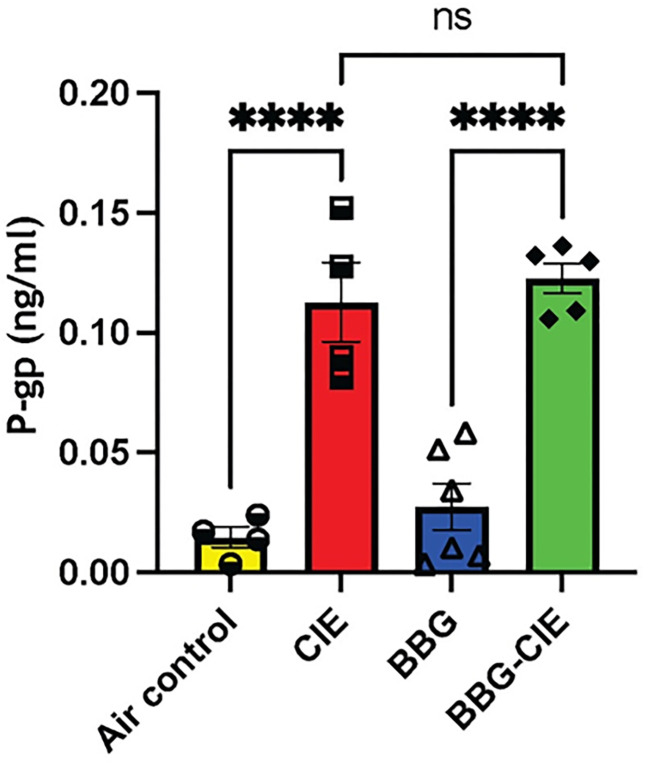
CIE exposure resulted in increased blood levels of P-glycoprotein (P-gp). The levels of P-gp after CIE exposure were analyzed by ELISA. Compared with those in air-control mice,plasma P-gp levels in CIE-exposed mice were significantly greater. Treatment with BBG had no effect on P-gp levels in BBG-treated CIE-exposed animals. One-way ANOVA followed by Tukey’s post hoc test was used for the statistical analyses. n = 5–7, mean ± SEM and ****p < 0.0001.

P-gp, an ATP-binding cassette subfamily B member 1 (ABCB1), plays a crucial role in BBB function and is involved in the efflux of toxic compounds back to the bloodstream [50]. It is only expressed on brain endothelium; therefore, it increase in blood indicate BBB injury. Plasma P-gp levels were significantly upregulated in CIE-exposed mice as compared to air-control mice. BBG treatment did not alter P-gp levels in CIE-exposed animals (Fig. 4).

### The increase in serum ATP and EV-ATP levels induced by CIE was reversed by pharmacologic or genetic P2X7R inhibition

**Figure 5:**
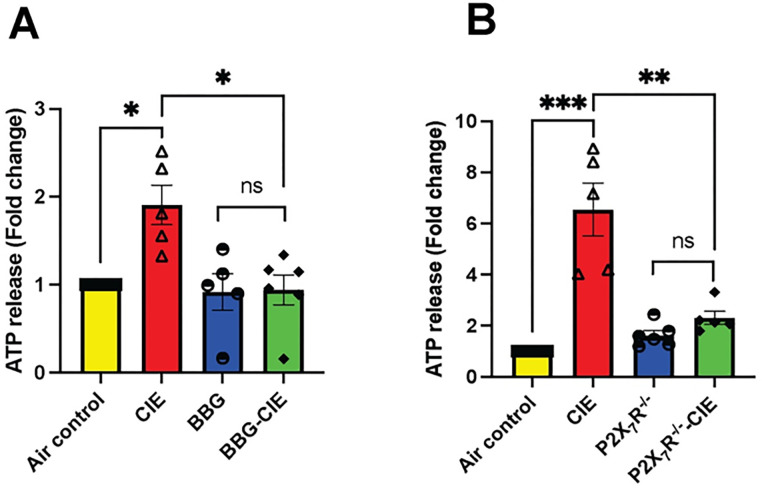
Serum ATP levels in CIE exposed animals were normalized by pharmacologic or genetic P2X7R inhibition. Serum ATP levels were lower in (A) BBG-treated CIE-exposed and B) P2X7R−/− CIE-exposed mice than in CIE-exposed mice. One-way ANOVA followed by Tukey’s post hoc test was used for the statistical analyses. N = 5–7, mean ± SEM and *p ≤ 0.05, **p < 0.01, and ***p < 0.0001; ns= nonsignificant. A two-tailed t test was used for the statistical analyses.

P2X7R overactivation increases ATP concentrations [51]. CIE exposure increased serum ATP levels by 2–6-fold in mice. BBG-treated CIE-exposed mice and P2X7R^−/−^ CIE-exposed mice exhibited significantly lower serum ATP levels than CIE-exposed mice (*p < 0.05, **p < 0.01, and ***p < 0.0001) (Fig. 5A and B).

**Figure 6:**
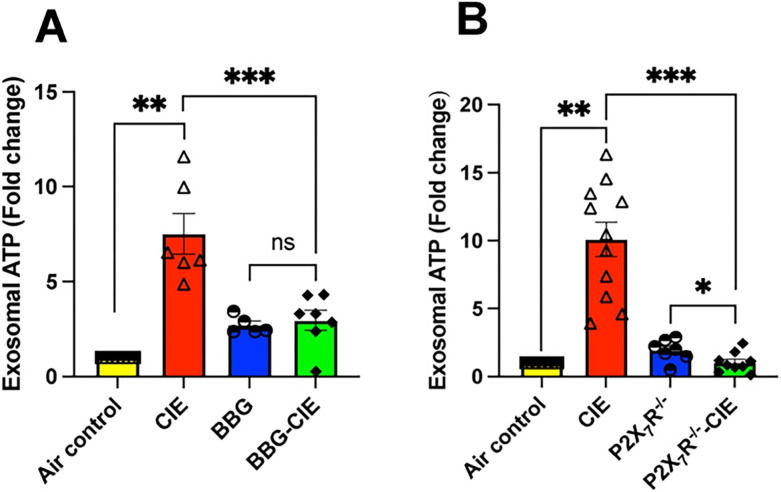
EV ATP content increased in CIE animals were diminished by pharmacologic or genetic P2X7R inhibition. The serum EV-ATP levels upregulated 7–10-fold by CIE were reduced in (**A)** BBG-treated CIE-exposed and (**B)** P2X7R^−/−^ CIE-exposed mice. One-way ANOVA followed by Tukey’s post hoc test was used for the statistical analyses. n = 5–7, mean ± SEM and *p £ 0.05, **p < 0.01 and ***p < 0.001, ns= nonsignificant; two-tailed t test was used for the statistical analyses.

Serum EV-ATP levels were upregulated 7–10-fold in CIE-exposed animals as compared to air control group. Serum EV-ATP levels were significantly downregulated in BBG-treated CIE-exposed mice and CIE-exposed P2X7R^−/−^ mice when compared to in CIE-exposed mice (*p < 0.05, **p < 0.01 and ***p < 0.001) (Fig. 6A and B).

### Increased EV numbers in CIE-exposed animals were reduced after P2X7R inhibition

**Figure 7:**
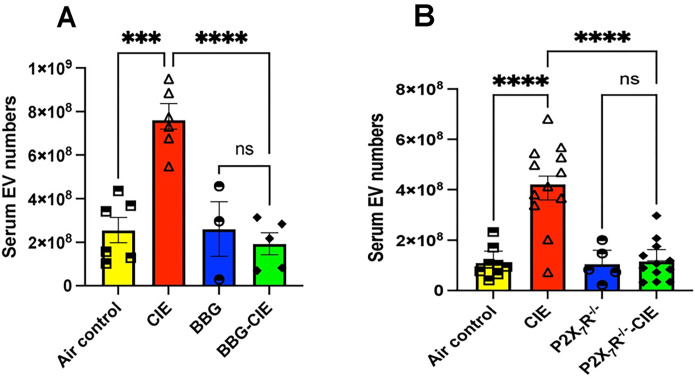
Genetic and pharmacologic P2X7R inhibition reduced EV numbers in CIE exposed mice. EV numbers were significantly lower in (**A)** BBG+CIE-exposed and (**B)** P2X7R^−/−^ CIE-exposed mice than in the respective CIE-exposed mice. One-way ANOVA followed by Tukey’s post hoc test was used for the statistical analyses; ***p < 0.001, **** p < 0.0001, and ns= nonsignificant (n = 5–7, mean ± SEM).

EVs play critical roles in intercellular communication and can transport various bioactive molecules [52]. Here, we investigated the impact of P2X7R inhibition followed during CIE exposure on EV generation and found a 2–4-fold increase in the number of EVs (Fig. 7A and B). We found a significant reduction (p < 0.0001) in the number of EVs in BBG-treated CIE-exposed and CIE-exposed P2X7R^−/−^ mice compared to their respective CIE-exposed controls.

### Genetic and pharmacologic P2X7R inhibition downregulated mtDNA copy numbers in EVs

**Figure 8:**
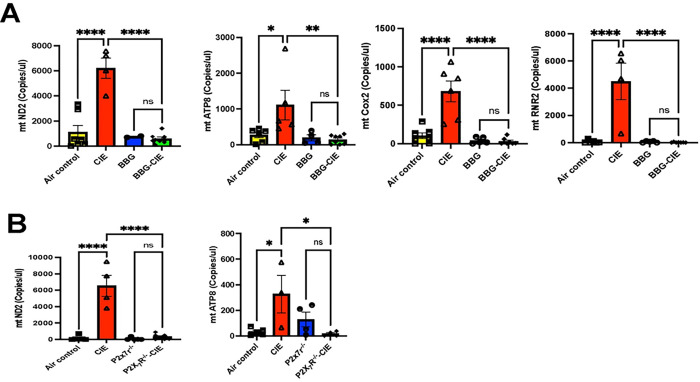
Genetic and pharmacologic P2X7R inhibition downregulated mitochondrial gene expression enhanced by CIE. Digital PCR was used to quantify the copy numbers of mtDNA present in EVs, and bar graphs represent the copy number of genes per microliter of DNA in the experimental groups. **(A)** P2X7R inhibition or **(B)** P2X7R^−/−^ knockout reduces mtDNA copy numbers in the EVs isolated from BBG-treated CIE exposed animals and CIE-exposed P2X7R^−/−^ compared to that of EVs from CIE-exposed animals. One-way ANOVA followed by Tukey’s post hoc test were used for the statistical analyses. mean ± SEM, * p ≤ 0.01, **p ≤ 0.001, **** p < 0.0001, and ns= nonsignificant. (n = 3–8)

We evaluated the three genes (*mt-ND2, mt-ATP8, mt-Cox2*) having crucial role mitochondrial respiration and other one (*mt-RNR2*) part of mitochondrial ribosome. We utilized digital PCR to quantify the copy numbers of mtDNA present in EVs. CIE-exposed mice showed significantly higher mtDNA level in EVs than air-control group which was significantly reduced in the EVs from BBG-treated-CIE-exposed mice. Copy numbers of *mt-ND2* and *mt-ATP8* were significantly lower in EVs from CIE-exposed P2X7R^−/−^ mice than in EVs isolated from CIE-exposed mice alone (Fig. 8A and B).

## Discussion

The present study sought to understand the role of P2X7R signaling in neuroinflammatory responses and BBB damage caused by CIE exposure in mice. We report, for the first time, that pharmacological blocking of P2X7R and genetic knockout of P2X7r may diminish CIE-induced BBB injury *in vivo.*

Chronic alcohol consumption alters the peripheral immune profile, signaling peripheral organs and brain microglia and astrocytes to release pro-inflammatory cytokines [53]. Upon CIE exposure, we noted significant increase in the serum levels of TNF-α, KC/GRO, IL-2, IL-1β, IFN-γ, and IL-5. Notably, increased levels of these cytokines in periphery are associated with alcohol use disorder in humans [54]. IFN-γ acts as potent inducer of TNF-α during neuroinflammation [55, 56]. The increased concentration of KC/GRO has been reported at the time of BBB damage [57]. Alcohol intoxication induced plasma IL-1β and IL-2 in rhesus macaques [58]. In the present study, levels of these cytokines were alleviated by pharmacological blockage and genetic knockout of P2X7R suggesting its crucial role in CIE-induced neuroinflammation (Fig. 2B). Similarly, Asatryan and collogues reported overactivation of the P2X7R and increased mRNA expression of proinflammatory cytokines IL-1B, TNF-α, and IL-6 in the mouse model of chronic EtOH exposure combined with high-fat diet [16, 59].

Studies have shown that BBB dysfunction amplifies neuroinflammation [9]. Brain microvessels serve as an excellent *ex vivo* model to study the BBB [48, 49, 60, 61]. Targeted blockade of P2X7R serves as a potential path to combat neuroinflammation [62–66]. In experimental autoimmune encephalomyelitis, BBG-dependent P2X7R antagonism resulted in decreased BBB damage with normalized levels of PDGFβR and claudin-5 and pro-inflammatory cytokines, IL-1β, IL-6, and TNF-α [67, 68]. Elevated CCL2 production via MCP-1/CCR2-mediated pathway was followed by P2X7R activation in brain [69]. P2X7R is involved in the caspase-1 activation leading to increased IL-1β and TNF-α levels, which causing apoptosis [70]. We found that BBG-induced P2X7R blockade resulted in a 2–50-fold downregulation of genes associated with inflammation (*cxcl1, il1b, cxcr5, tnf, il6, sele, cxcl2,* and *ccl2),* apoptosis *(fasl, il3, bcl2, casp1, and il7*), vasodilation (*ednra and agtr1a*), and platelet activation (*serpine1, selp, timp1, il11, f2r, and pdgfra*) (Table 1, Fig. 2A). These changes in gene expression in BBG-treated-CIE-exposed mice underscore the significant impact of P2X7R inhibition on the transcriptional landscape within brain microvessels during chronic EtOH exposure.

In chronic inflammation and neurodegenerative diseases, enhanced P2X7R shedding has been observed [71–73]. Additionally, *in vitro* dendritic cell P2X7R stimulation with ATP can trigger shedding of microvesicles carrying the P2X7R itself, suggesting a regulatory role of P2X7R signaling in its own shedding process [74]. Earlier, we showed that *in vitro* treatment of lung epithelial cells with EtOH increased P2X7R shedding [18]. In this study, we observed a substantial increase in the circulatory P2X7R levels in CIE-exposed animals, which was significantly reduced pharmacologic or genetic P2X7R inhibition (Fig. 3A and B). The observed results add to the growing body of evidence, implicating P2X7R shedding in the regulation of P2X7R signaling and activity.

P-glycoprotein (P-gp) is an efflux transporter with a crucial role in the transport of substances across the BBB. Chronic alcohol exposure significantly increases P-gp mRNA and protein expression *in vitro* [75]. TNF-α and endothelin-1 exposure also stimulates P-gp expression [76]. We found a significant increase in P-gp levels in the blood of CIE-exposed mice reflecting BBB injury. However, treatment with BBG did not alter P-gp levels in CIE-exposed animals, suggesting that P2X7R signaling may not regulate P-gp expression (Fig. 4). More recently, Arnaud-Sampaio and colleagues have shown that P2X7R isoform B has higher efflux activity than P2X7R A, which may be mediated by P-gp and other ABC transporters [77].

P2X7R are ATP-gated cation channel receptors and undisputedly serve as gatekeepers of inflammation [63, 78]. P2X7R-dependent ATP release contributes to increased extracellular ATP levels in osteoclast and osteoblast cultures, highlighting autocrine/paracrine role of P2X7R signaling [79]. Studies have highlighted the importance of P2X7R-mediated ATP release in initiating and amplifying inflammatory responses in the CNS and peripheral tissues [80]. Similarly, we found increased serum ATP level in CIE-exposed mice. The BBG-treated or P2X7R^−/−^-CIE-exposed mice exhibited significantly lower serum ATP levels compared to CIE-exposed mice (Fig. 5). This suggests that inhibition or genetic knockout of the P2X7R reduces eATP release, a neuroinflammatory messenger, mitigating neuroinflammatory responses associated with chronic alcohol exposure [81].

Several studies have reported that P2X7R stimulation by ATP triggers EV shedding with significant change in their size [20, 21]. Moreover, EV cargo composition in various cell types, including immune cells and neurons is influenced by P2X7R stimulation by ATP [22–24, 82]. In the context of chronic alcohol exposure, limited research has explored the role of P2X7R in EV regulation and its implications for alcohol-induced pathology. Pfeiffer and colleagues have noted that ATP-dependent P2X7R activation results in P38-MAPK-facilitated cytoskeletal restructuring, leading to EV release [83]. We found a drastically increased number of EVs in CIE-exposed mice, which was significantly lowered in both BBG-treated and P2X7R^−/−^ CIE-exposed mice (Fig. 7). These data indicate a potential role of P2X7R in EV biogenesis and secretion due to alcohol exposure *in vivo.* The mechanism of increasing EV generation and their content changes are of considerable interest to investigate.

Interestingly, Ibáñez and collogues have reported EtOH-induced EV secretion with significant alterations in lipid metabolism and EV enrichment with inflammatory-related proteins and miRNAs in BV2 microglia and astrocytes [25, 84]. Studies have reported changes in EV composition after alcohol exposure in liver and lung cells [85, 86]. EV-mediated ATP signaling plays an important role in regulating inflammatory responses and immune cell activation [87, 88]. Along with other components, ATP itself is present in released EVs [23, 89]. Our study showed reduced serum EV-ATP levels following P2X7R inhibition or knockout in CIE-exposed mice (Fig. 6), potentially mitigating the pro-inflammatory and pathological effects associated with chronic alcohol exposure.

EVs can cross BBB, carry exchange between CNS and blood, and regulate neuroinflammation [90–92]. P2X7R overactivation leads to mitochondrial damage, causing the release of mitochondrial content, including Ca^2+^, ATP, and mtDNA into the extracellular environment. [17, 93]. Chronic alcohol exposure of hepatocyte causes release of EVs, containing mtDNA fragments, which act as DAMPs with proinflammatory properties, activating autocrine and paracrine signaling pathways [28]. Of note, EtOH-induced mtDNA damage is known to be involved in the release of exosomes enriched with damaged mtDNA *in vitro* [94]. Recent reports have demonstrated the presence of mtDNA fragments in hepatocyte-derived EVs after EtOH-exposure *in vivo* and in patients with fatty liver conditions [26, 27]. Our prior study showed that P2X7R inhibition reduces mtDNA copy numbers in EVs from EtOH-treated lung epithelial cells *in vitro* [18]. In this study, we report a significant increase in mtDNA copy numbers in isolated EVs from CIE-exposed animals (Fig. 8A, B), whereas P2X7R inhibition or knockout significantly reduced mtDNA content in CIE-exposed mice. It is known that cytosolic mtDNA-mediated NLRP3 inflammasome activation is associated with caspase-1 activation and IL-1β/IL-18 production [95, 96]. The increased expression of caspase-1 and IL-1β (Fig. 2A, Table 1) in isolated brain microvesicles further supports this observation. Additionally, mtDNA is known to activate TLR9-MyD88 downstream signaling, causing NF-κB activation, which triggers the production of pro-inflammatory cytokines and chemokines [97, 98]. Increased mtDNA concentration in isolated EVs and significantly increased gene expression of *tnf-α, il-6, il-1β, cxcl1, cxcl2,* and *cxcr5* from EtOH-exposed groups corroborate these findings (Fig. 2A, Table 1). In earlier studies, the presence of mtDNA in EVs was detected using gene-specific probes for mt-ND2, mt-ATP8, mt-Cox2, and mt-RNR2 [46, 99]. These genes are involved in mitochondrial respiration [100]. In the present study, significantly increased mtDNA concentration in EVs and proinflammatory changes in the periphery and BBB of CIE-exposed group suggest that the mtDNA carried by EVs acts as DAMP and may cause an exacerbation of neuroinflammation [99, 101].

Our observations imply that P2X7R signaling may play a role in regulating EV release and their cargo contents. While the specific mechanisms underlying P2X7R-mediated regulation of mitochondrial stress and escape of mtDNA in EVs remain to be fully elucidated, our findings suggest a potential link between P2X7R signaling, mitochondrial dysfunction, and EV dynamics in the context of chronic alcohol exposure and neuroinflammation. Future studies of the mechanism of EV release, link to P2X7R activation, and cross organ communication by EVs may pave the way to future treatment interventions.

## Conclusion

The present study delved into understanding the impact of P2X7R signaling on neuroinflammatory responses and BBB injury induced by CIE exposure. Blockade of P2X7R channels resulted in reduced eATP release, enhanced BBB function, and downregulation of genes associated with inflammation, apoptosis, vasodilation, and platelet activation, underscoring the critical role of P2X7R in CIE-induced neuroinflammation. Furthermore, inhibition or genetic knockout of P2X7R led to altered EV dynamics, such as reduced quantity and eATP and mtDNA levels, suggesting a potential regulatory role of P2X7R signaling in mitigating chronic alcohol-induced pro-inflammatory effects associated with EVs. These findings contribute to understanding the complex interplay between P2X7R signaling, peripheral and neuro-inflammation, BBB integrity, and circulating EV biology in the context of *in vivo* chronic alcohol exposure.

## Figures and Tables

**Table 1:** Fold changes in the expression of genes involved in inflammation, apoptosis, vasodilation, and platelet activation in the brain microvessels of CIE-exposed animals treated with or without BBG. The observed values are normalized against the air control group.

		Fold regulation	
Gene	Gene	CIE-Exposed	BBG-CIE
*Nppb*	Natriuretic peptide type B	3.04	−51.23
*Pig*	Plasminogen	1.25	−48.29
*Fasl*	Fas ligand (TNF superfamily, member 6)	2.48	−45.54
*Cxcl1*	Chemokine (C-X-C motif) ligand 1	2.00	−36.23
*Cxcr5*	Chemokine (C-X-C motif) receptor 5	1.45	−35.94
*Mmp1a*	Matrix metallopeptidase 1a (interstitial collagenase)	6.25	−25.97
*Il3*	Interleukin 3	3.90	−20.45
*Il11*	Interleukin 11	2.33	−15.05
*Tnf*	Tumor necrosis factor	2.49	−15.18
*Agtrla*	Angiotensin II receptor, type 1a	2.46	−12.56
*Il6*	Interleukin 6	3.59	−12.50
*Sele*	Selectin, endothelial cell	3.16	−11.05
*Cxcl2*	Chemokine (C-X-C motif) ligand 2	5.86	−9.42
*Ccl2*	Chemokine (C-C motif) ligand 2	2.72	−8.66
*Il7*	Interleukin 7	2.97	−7.64
*Tymp*	Thymidine phosphorylase	1.85	−4.82
*Serpine1*	Serine (or cysteine) peptidase inhibitor, clade E, member 1	1.67	−4.54
*Pgf*	Placental growth factor	1.67	−2.86
*Selp*	Selectin, platelet	1.62	−5.52
*Il1b*	Interleukin 1 beta	1.49	−5.49
*Ccl5*	Chemokine (C-C motif) ligand 5	1.46	−3.08
*Tmp1*	Tissue inhibitor of metalloproteinase 1	1.28	−4.75
*Bcl2*	B-cell leukemia/lymphoma 2	1.16	−2.75
*Ednra*	Endothelin receptor type A	1.88	−2.96
*Casp1*	Caspase 1	2.69	−1.54
*Mmp9*	Matrix metallopeptidase 9	1.58	−1.59
*Tfpi*	Tissue factor pathway inhibitor	2.57	−1.12
*Kit*	Kit oncogene	1.46	−1.66
*Angpt1*	Angiopoietin 1	1.45	−1.39
*F2r*	Coagulation factor II (thrombin) receptor	2.49	−1.85
*Plau*	Plasminogen activator, urokinase	1.51	−1.08
*Pdgfra*	Platelet derived growth factor receptor, alpha polypeptide	1.16	−1.09

## Data Availability

All data generated or analyzed during this study are included in this published article or its supplementary information files.

## Abbreviations

BBB: Blood–brain barrier
EV: Extracellular vesicle
CIE: chronic intermittent ethanol (CIE)
BBG: Brilliant Blue G
BEC: Blood ethanol concentration
eATP: Extracellular ATP
P7X7R: Purinergic receptor P2X7
DAMPs: Damage-associated molecular patterns
NLRP3: Nod-like receptor pyrin domain containing 3
EtOH: Ethanol
BMVECs: Brain microvascular endothelial cells
mtDNA: Mitochondrial DNA
mt-ATP-8: Mitochondrially encoded ATP synthase membrane subunit 8
mt-ND2: NADH dehydrogenase 2
mt-COX2: Cytochrome c oxidase subunit II
mt-RNR2: 16S ribosomal RNA
HBSS: Hank’s balanced salt solution
P-gp: P-glycoprotein
KC/GRO: Keratinocyte chemoattractant (KC)/human growth-regulated oncogene (GRO)
TNF-α: Tumor necrosis factor alpha
IFN-γ: Interferon gamma
IL-1β: Interleukin 1 beta
IL-2: Interleukin-2
IL-4: Interleukin-4
IL-5: Interleukin-5
IL-6: Interleukin-6
IL-10: Interleukin-10
IL12p70: Interleukin-12 p70
